# Laparoscopic Technique for Serial Collection of Para-Colonic, Left Colic, and Inferior Mesenteric Lymph Nodes in Macaques

**DOI:** 10.1371/journal.pone.0157535

**Published:** 2016-06-16

**Authors:** Jeremy Smedley, Rhonda Macalister, Solomon Wangari, Mercy Gathuka, Joel Ahrens, Naoto Iwayama, Drew May, Debbie Bratt, Megan O’Connor, Paul Munson, Michael Koday, Jeff Lifson, Deborah Heydenburg Fuller

**Affiliations:** 1 Division of Primate Resources, Washington National Primate Research Center, University of Washington, Seattle, Washington, United States of America; 2 Oregon National Primate Research Center, Oregon Health Sciences University, Beaverton, Oregon, United States of America; 3 Laboratory Animal Sciences Program, Leidos Biomedical Research, Inc., Frederick National Laboratory Frederick, Maryland, United States of America; 4 Division of AIDS Research, Washington National Primate Research Center, Department of Microbiology University of Washington Seattle, Washington, United States of America; 5 AIDS and Cancer Viruses Program, Leidos Biomedical Research, Inc., Frederick National Laboratory, Frederick, Maryland, United States of America; Harvard Medical School, UNITED STATES

## Abstract

Unlike peripheral lymph nodes (PLN), the mesenteric lymph nodes (MLN) draining the gastrointestinal (GI) tract are exposed to microbes and microbial products from the intestines and as such, are immunologically distinct. GI draining (MLN) have also been shown to be sites of early viral replication and likely impact early events that determine the course of HIV infection. They also are important reservoir sites that harbor latently-infected cells and from which the virus can emerge even after prolonged combination antiretroviral therapy (cART). Changes in the microbial flora and increased permeability of the GI epithelium associated with lentiviral infection can impact the gut associated lymphoid tissue (GALT) and induce changes to secondary lymphoid organs limiting immune reconstitution with cART. Nonhuman primate models for AIDS closely model HIV infection in humans and serial sampling of the GALT and associated secondary lymphoid organs in this model is crucial to gain a better understanding of the critical early events in infection, pathogenesis, and the role of immune responses or drugs in controlling virus at these sites. However, current techniques to sample GI draining (MLN) involve major surgery and/or necropsy, which have, to date, limited the ability to investigate mechanisms mediating the initiation, persistence and control of infection in this compartment. Here, we describe a minimally invasive laparoscopic technique for serial sampling of these sites that can be used with increased sampling frequency, yields greater cell numbers and immune cell subsets than current non-invasive techniques of the GALT and reduces the potential for surgical complications that could complicate interpretation of the results. This procedure has potential to facilitate studies of pathogenesis and evaluation of preventive and treatment interventions, reducing sampling variables that can influence experimental results, and improving animal welfare.

## Introduction

The lymph nodes (LN) draining the gastrointestinal (GI) tract are often generically called the mesenteric lymph nodes (MLN). These include a number of distinct nodes at varying proximity to the intestines, including the para-colonic, left colic, superior and inferior mesenteric lymph nodes among others. Dendritic cells and macrophages from the intestines traffic to the MLN and activated T and B cells from the MLN traffic to the lamina propria of the intestine creating a compartmentalized immune response that can be distinct from systemic immune responses [1]. In addition to playing major roles in conditions such as inflammatory bowel disease, Crohn’s disease, and food allergies [2], these immunologically unique sites play important roles in HIV and AIDS.

Approximately 1.2 million people in the United States were living with HIV infection in 2012 [3], with gay, bisexual, and other men who have sex with men (MSM) disproportionately affected. In 2013, MSM accounted for 81% of the estimated 37,887 HIV diagnoses among males aged 13 years and older [4]. Even with early initiation of combined antiretroviral therapy (cART) suppression, persistent viral reservoirs are still established [5–6] and often mediate the rapid viral rebound that typically occurs after cessation of cART administration [7]. The MLN have been identified as an important reservoir site for the virus [8–9] and have even been suggested to be the primary reservoir site [10–12]. HIV infection causes mucosal epithelial damage with translocation of microbial products from the GI tract contributing to local and systemic immune activation. The resulting alterations of lymph node architecture, including fibrotic changes with damage to the fibroblastic reticular cell network (FRCn) of LN lead to T cell depletion and limit immune reconstitution after initiation of cART, findings that have been recapitulated in studies of SIV infected macaques [13–15]. Typically the MLN remove enteric microbes and microbial products before a significant systemic response occurs resulting in compartmentalization of the response to the GI system and associated lymphoid organs. However HIV infection disrupts both the intestinal barrier and local immune responses. The GI draining LN are the first to encounter translocated microbial products and thus are a critical site for evaluation of these effects through the course of infection, including during cART suppression [14–15].

Recently, we demonstrated in a macaque model of rectal HIV transmission that after intrarectal inoculation, viral dissemination likely occurs via draining lymphatic pathways that include the para-colonic, left colic, and inferior mesenteric LN [16]. As the first secondary lymphoid organ to encounter the virus, evaluation of the effects of HIV/SIV infection and interventions in the MLN are critical for studying the early events that shape the course of infection and the impact of vaccines and antivirals.

Current techniques to evaluate the effect of SIV or SHIV infection on MLN or GALT in living macaques involve noninvasive imaging [17], endoscopic colonic or duodenal/jejunal biopsies or intestinal resections and/or MLN collection that entail major abdominal surgery, resulting in a very limited number of sampling time-points and increased risk of complications to the animals and potential adverse impact on experimental results. Each of these approaches have significant drawbacks, including insufficient sample size or number of time-points to study the effects of infection, vaccines or therapies on immune cells or the viral reservoir. We therefore sought to develop a minimally invasive laparoscopic technique that would allow for more frequent serial sampling with minimal impact to the animals to evaluate effects of vaccines or therapies on immune responses, early events post transmission, viral replication and latency reversal in these critical sites.

## Material and Methods

### Nonhuman primates

In vivo primate studies were conducted at 2 sites; National Cancer Institute, National Institutes of Health (Bethesda, MD) and at the Washington National Primate Research Center (Seattle, WA). Animals were housed and cared for in Association for the Assessment and Accreditation of Laboratory Animal Care standards (AAALAC) accredited facilities, and all animal procedures were performed according to protocols approved by the Institutional Animal Care and Use Committees of the University of Washington and the National Cancer Institute. All animals were pair housed in stainless steel cages meeting the USDA requirements for space based on their body weights, were feed (Purina 5046 monkey chow) twice daily based on their individual needs and water was available ad libitium. Temperatures were 75± 2°F, and humidity was 30–70%. Environmental enrichment consisted of toys in and on the cage, foraging devices, mirrors, radios, and televisions. All surgeries were survival procedures and no animals were euthanized as part of the described procedures.

### Laparoscopic procedures

**Fig 1:**
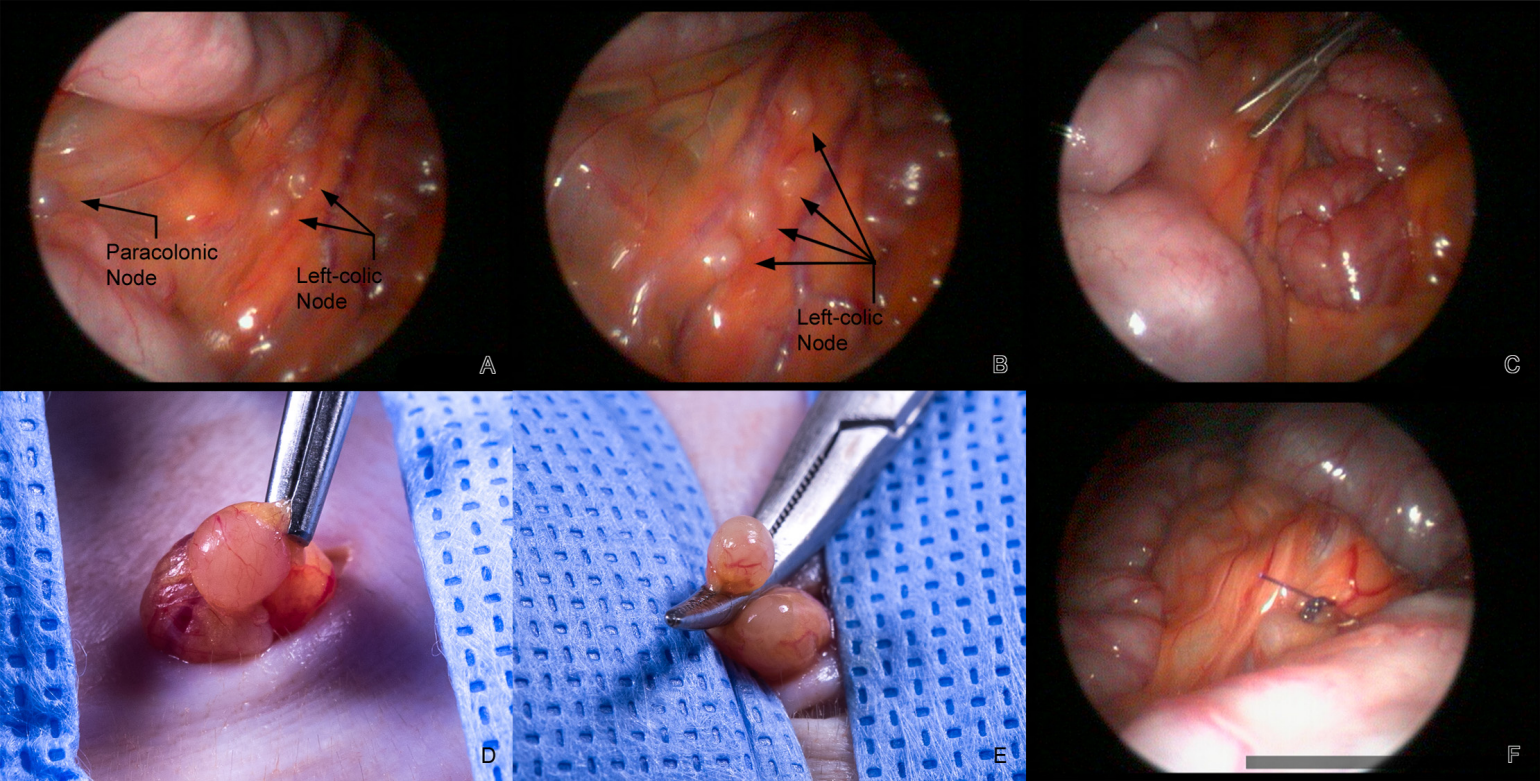
**A**. Laparoscopic view of a paracolonic and two left-colic nodes draining a portion of the descending colon. **B**. Four left colic nodes draining a different portion of the descending colon. **C**. Use of the Maryland forceps to grasp the mesentery adjacent to a left-colic node prior to externalization. **D**. Externalized node held with a rat tooth forceps. **E**. Externalized node clamped with a mosquito hemostat prior to ligation. **F**. 4–0 PDS at ligature site in the abdomen.

Animals were anesthetized using 10–15 mg/kg Ketamine and 15-25ug/kg dexmedetomidine, intubated and maintained on 0.5–2% isoflurane. The abdomen was clipped and asceptically prepped using alternating chlorhexadine and alcohol following standard techniques. Surgeons performed a surgical scrub and wore sterile gowns and gloves. All instruments were steam autoclaved prior to use. Laparoscopy was performed using Dyonics 460P-3 Chip Camera, Dyonics 300XL Xenon Light Source and Dyonics Access 40L Insufflator. The animal was placed at an approximately 30–45 degree angle part way between right lateral and dorsal recumbency. A 20–30 degree Trendelenburg position was also used to improve visualization and positioning of the descending colon. After insufflation with a Veress needle to 8–10 mmHg a ~5mm incision was made for port placement in the left cranial abdominal quadrant. A 2.7mm 0 degree scope was inserted through this port to allow examination of the abdomen and determination of the position for the placement of the second port. A second ~5mm incision was made just left of the midline at about the level of the umbilicus for instrument placement. A 5mm blunt probe was used to remove the omentum from around the intestines and to position the descending colon along the left abdominal wall to allow visualization of the medial side of the mesenteric attachment. Paracolonic, left colic and inferior mesenteric lymph nodes were visualized and a Maryland ratchet forcep was used to grasp the mesentery near the node and externalize it. The node was then dissected and ligated with 4–0 PDS before removal (Fig 1). This process was repeated up to two additional times at each time point for a total of up to 3 nodes collected depending on study requirement. The abdomen was then visualized with the laparoscope to ensure that there were no complications from the procedure. The abdomen was deflated and the abdominal wall and skin closed in a two layer closure using 4–0 PDS. The entire process from incision to closure took 15–40 minutes depending on the size and body condition of the animal.

Animals received a local block of the incisions with Marcaine (0.5%) and buprenorphine (0.3mg/kg) and ketoprofen (3mg/kg) both given IM or sustained release buprenorphine (0.2 mg/kg) SQ and meloxicam (0.2 mg/kg) IM were given for postop pain relief. All animals were assessed postoperatively by experienced primate veterinarians.

Colonic biopsies (18) for comparison were collected from the descending colon with a flexible endoscope using 1.9mm cup biopsy forceps.

### Cell Isolation and Quantification

After removal, lymph nodes were placed in RPMI. Supernatants were filtered through a 70 um filter, pieces of tissue were then crushed on the filters, and were washed twice with R10 media (RPMI supplemented with 10% heat-inactived fetal calf serum). Cells were mixed with trypan blue at a 1:1 ratio and were counted on a Cellometer Auto T4 (Nexcelom Bioscience, Lawrence, MA). Viability and cell yield means +/- the standard error of means (SEM) were calculated and compared between colon and MLN samples by a Wilcoxon matched-pairs signed rank test using GraphPad Prism software.

### Flow Cytometry

Multicolor flow cytometric analysis was performed on cells isolated from the MLN and colon. Cells were stained with the following Abs: anti-CD3 Alexa Fluor 700 (clone SP34-2, BD Pharmingen, San Jose, CA); anti-CD8 APC-Cy7 (clone RPA-T8, BD Pharmingen); anti-CD20 BV570 (clone 2H7, BioLegend, San Diego, CA); anti-CD4 BV605 (clone OKT4, BioLegend); anti-CD45 PE-CF594 (clone D058-1283, eBioscience, San Diego, CA); anti-CD14 BV785 (clone M5E2, BioLegend); anti-IL-17 (clone eBio64CAP17, eBioscience), IFNγ (clone B27, BD Pharmingen), TNFα (clone MAb11, BD Pharmingen), and CD107a (clone eBioH4A3, eBioscience). All cells were stained with the Live/Dead Fixable Aqua Dead Cell Stain Kit (Thermo Fisher Scientific, Grand Island, NY) to exclude dead cells from the analysis. Samples were permeabilized and fixed using CytoFix/Perm Kit (BD Pharmingen) and stained for intracellular cytokines. For intracellular cytokine staining (ICS), cells were stimulated with PMA (10ng/ml; Sigma Aldrich) and Ionomycin (1μg/ml, Thermo Fisher Scientific) for 10–14 hours with brefeldin A (1μg/ml; Sigma Aldrich). Flow cytometric acquisition was performed on a BD LSRII cytometer using FACs Diva software (BD Pharmingen) and analysis of the data was performed using FlowJo software (Tree Star, Ashland, OR). Differences in cell subsets and immune responses measured in the colon and MLN samples were compared by a paired t-test (Mann-Whitney) using GraphPad Prism software.

## Results

This procedure has been performed on 62 rhesus macaques with 86 total collections. For animals that were not obese the success rate in obtaining samples was 100% and there were no complications including in animals that were infected with SIV or SHIV. The procedure has been performed on thrombocytopenic animals as well and as all vessels are ligated there was no significant hemorrhage. In obese macaques the success rate has been 50% (2/4) with visualization of lymph nodes through excessive mesenteric fat being the main obstacle. These animals often exhibit friability of the mesentery as a result of the adipose tissue as well, making exteriorization difficult. However, collection was successfully performed on animals where collection was not possible initially due to obesity after they were returned to a normal body condition. This procedure was successfully performed (13/14) in animals that had undergone prior major abdominal surgery, such as intestinal resection and anastomosis, with the only issue being the need to work around any adhesions that had formed to the abdominal wall. The one failure was in an obese animal.

Up to three longitudinal laparoscopic collections have been performed on individual animals (n = 14) with up to 3 MLNs collected at each time point with no increase in complications or changes in the success rate. As seen for peripheral lymph nodes, significant changes in size of these nodes have been observed over the course of SIV/SHIV infection with larger MLN typically seen during acute infection. Fig 1 shows representative images of the procedure and isolation of a MLN in a rhesus macaque that was infected 6 weeks prior with SIV. After insufflation a blunt probe was used to remove the omentum and manipulate the intestines to permit visualization of the medial aspect of the colonic mesentery and associated lymph nodes. Nodes to be retrieved were selected based on the size, proximity to one another and the ability to exteriorize the node. The mesentery near the node was grasped with a Maryland forceps, the abdomen was deflated, and the node externalized. The node was then ligated with 4–0 PDS and after checking the site for bleeding, the mesentery was returned to the abdomen.

**Fig 2:**
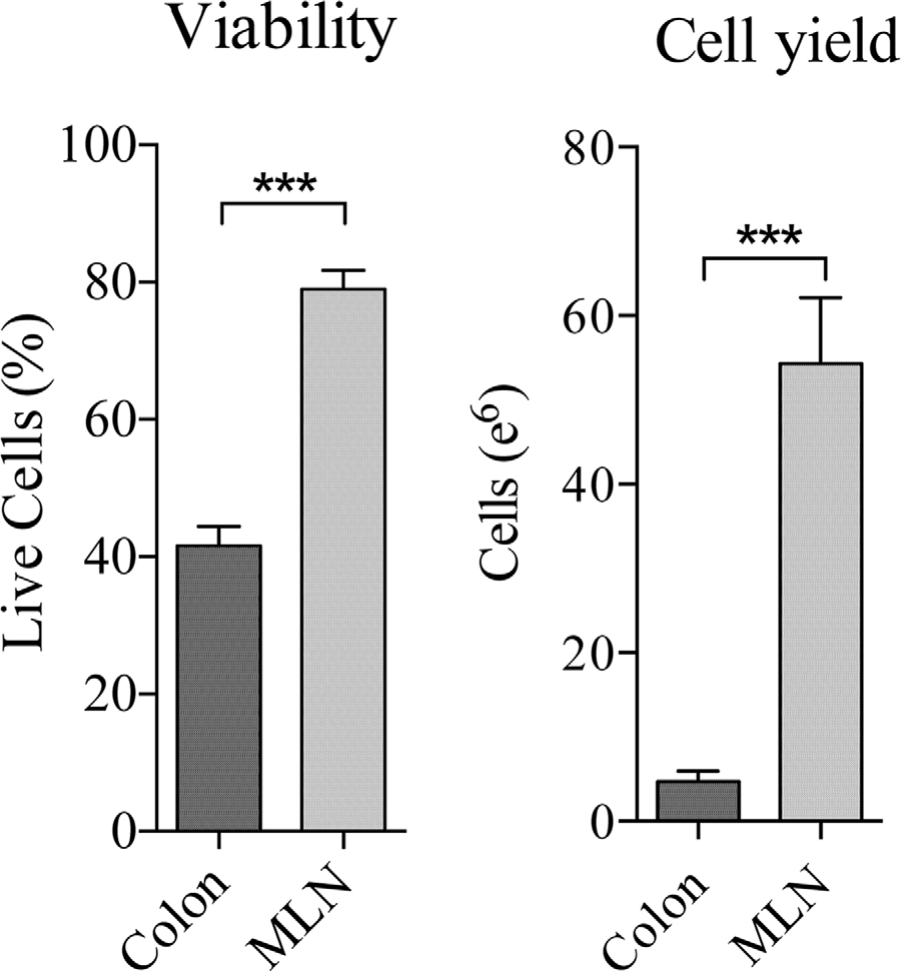
Viability and cellular yield of lymph nodes (2–3) collected via laparoscopy and colonic samples (18) collected via endoscopy at the same time points. *** p <0.0001

Endoscopic biopsies are currently the primary non-surgical method used to collect tissues and cells for analysis of the effects of infection, vaccination or therapies on the GALT. Similar to the MLN laparoscopy procedure proposed here, this approach offers greater frequency in sampling than surgical methods but often yields cell numbers (1–2 X 10^6^) that are too low to support a broad analysis of immune responses in the GALT including analysis of cell subset frequency, T and B cell specificity and function. This is due, in part, to the small amount of tissue that can be collected and a rigorous enzymatic processing procedure to separate the immune cells from the tissue that reduces cell viability and requires significant personnel effort. In contrast, LN mononuclear cells (LNMC) are typically isolated by a simpler, more rapid procedures that involves passing the tissue through a mesh screen. To determine relative cell yield and viability obtained from typical procedures of each type, the number of immune cells isolated from 2–3 MLNs collected via laparoscopy was compared to that from 18 colonic mucosa samples taken via endoscope at the same time point (n = 14 animals). The results in Fig 2 show that both the total cell yields and viability of cells isolated from mesenteric lymph nodes (MLN) were much greater than that of conventional colon endoscopic biopsies and that cellular yields from MLN were over 10-fold greater than from colon biopsies (P <0.0001).

**Fig 3:**
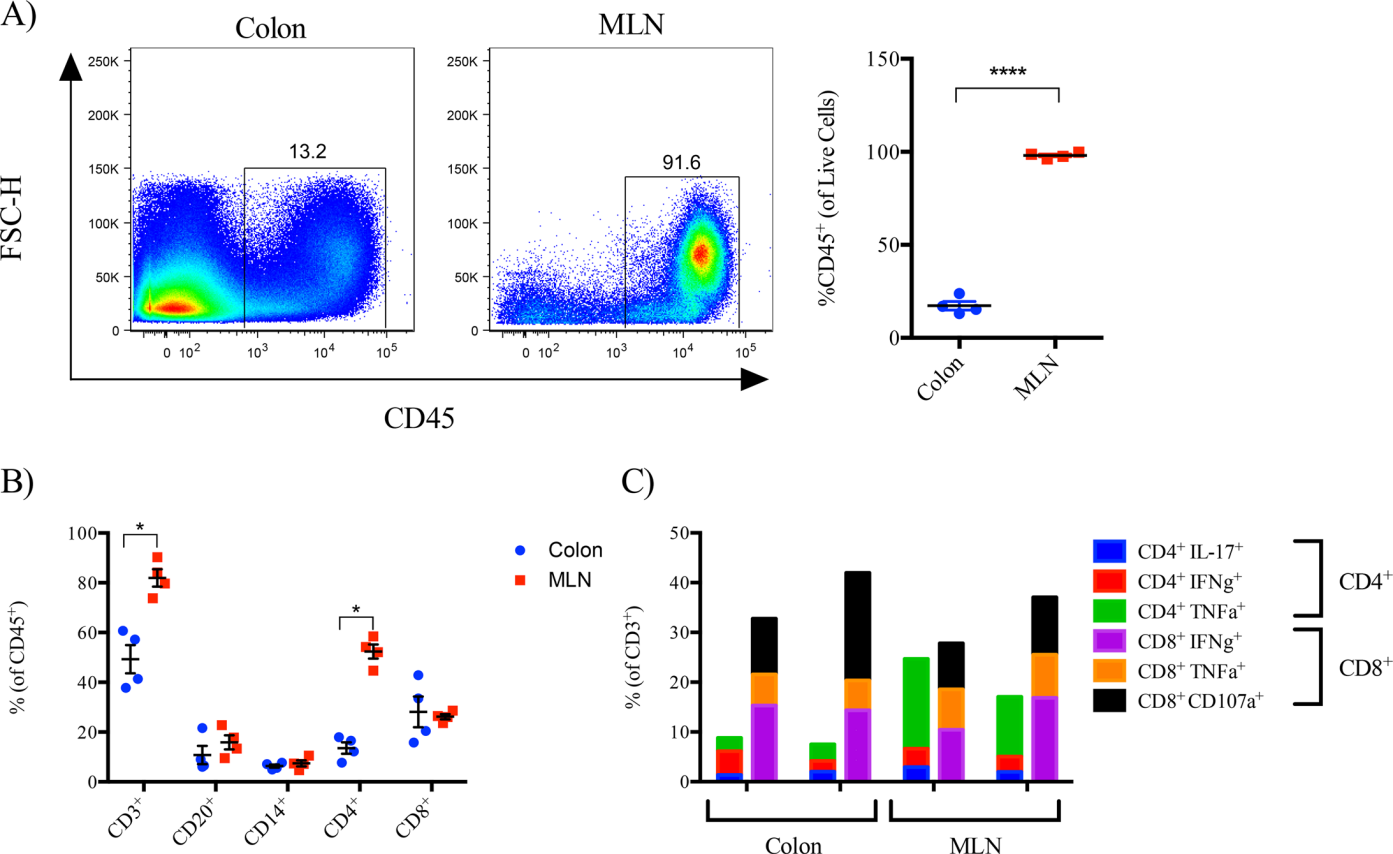
Cells were isolated concurrently from the colon and MLN of ART-treated, SIV-infected (8wpi) macaques and analyzed by flow cytometry. A. Flow cytometry plots and frequency of CD45+ cells of live cells. B. Frequency of CD3, CD20, CD14, CD4, and CD8 cellular subsets of live/CD45+ cells. C. Frequency of live/CD45+/CD3+ /(CD4+ or CD8+) cells with cytokine (IL-17, IFNγ, TNFα) or cytolytic (CD107a) effector function following in vitro stimulation with PMA and Ionomycin.

To compare immune cell subsets in the colon and MLN, cells isolated were isolated concurrently from the MLN and colon of SIV-infected macaques (at 8 weeks post-infection) that had received ART therapy starting at 6 weeks post-infection and analyzed by flow cytometery. The proportion of CD45^+^ lymphocytes was significantly higher in the MLN when compared to that observed in the colon (Fig 3A). Similar frequencies of CD20^+^, CD14^+^, and CD8^+^ cells were observed between the MLN and colon (Fig 3B) whereas frequencies of CD3^+^ T-cells and in particular, the CD4^+^ subset, were significantly higher in the MLN when compared to the colon (Fig 3B). To determine if T cells in the MLN and colon exhibited functional differences, intracellular cytokine staining (ICS) was performed on cells isolated from the MLN and colon. Pairwise comparisons were difficult due to low cell yield from the colon. However, results shown in Fig 3C show similar frequencies of most cytokine expressing T-cells were observed in the colon and MLN, with the exception that the MLN exhibited a higher frequency of TNFα producing CD4^+^ T-cells following *in vitro* stimulation with PMA and Ionomycin (Fig 3C).

## Discussion

We have developed a rapid, minimally invasive technique for sampling the paracolonic, left colic, and inferior mesenteric LN in macaques and demonstrated high rates of success with no adverse events, even with serial sampling. Paracolonic and left colic LN were easily retrievable in all animals. Inferior mesenteric LNs were more challenging in larger animals given the limited mobility of these nodes in the mesentery and the greater distance necessary to achieve externalization of the node. Complications were not observed even with serial collections, collections after previous laparotomies, and collections on animals experiencing immunosuppression and/or thrombocytopenia. Obesity appeared to be the main contraindication as it results in poor visualization of lymph nodes and friability of the tissue, increased bleeding, and interference with externalization of the node.

Viability of cells isolated from mesenteric lymph nodes (MLN) was nearly double that of conventional colon endoscopic biopsies (Fig 2). Additionally, cellular yields from MLN were over 10-fold greater than colon biopsies and contained significantly higher frequencies of CD45^+^ lymphocytes (Fig 3A). While endoscopic GI biopsies can provide valuable information, the low yield of viable cell yields substantially limits the scope of analysis. In contrast, our results show that a novel laparoscopic MLN biopsy procedure provides a minimally invasive alternative for survival sampling of the compartmentalized GI immune system that yields sufficient cell numbers to support a broader range of virological and immunological analyses. Higher MLN cellular yields, for example would allow for detection of viral RNA/DNA, immune-phenotyping and a broader characterization of mucosal immune responses including more extensive characterization of the function, specificity and avidity of SIV specific B and T-cellular responses by current state-of-art assays for analyzing these responses such as IFNγ and B cell enzyme linked immunosorbent (ELISpot) assays and intracellular cytokine stain (ICS) assays.

Previous studies have demonstrated that MLN represent GI cell lymphoid populations [18–19]. Our results similarly show that the MLN has similar cell subsets and immune response profile as the GI tract but we also observed distinct differences. In particular, the MLN exhibited an enriched frequency of CD4^+^ T cells when compared to the colon (Fig 3C) and a higher frequency of TNFα responses that may explain, in part, why the MLN is a primary reservoir for HIV/SIV. Although not measured in this study, follicular dendritic cells (FDC) and follicular T helper cells found in lymph nodes are unlikely to be found in many endoscopic pinch samples. Follicular aggregates found in endoscopic mucosal biopsies are rudimentary structures that are quite distinct from lymph nodes. These aggregates are found in a variable but often low percentage of arbitrary pinch biopsies and are always mixed with populations of cells from the lamina propria making lymph node the preferred sample for analysis of lymphoid follicles. Furthermore, longitudinal laparoscopic collection of MLN allows serial evaluation of both a primary reservoir site and in many cases the first secondary lymphoid organ draining translocated microbial products from the GI tract with associated immune activation and pathologic changes, such as inflammatory fibrosis.

In summary, we report a new technique that allows for serial sampling of the MLN with no adverse effects. Furthermore, we show this approach provides several advantages when compared to the more commonly employed endoscopic biopsy including higher cell yield and viability and a more representative immune cell subset for evaluation of previously identified pathologic changes, such as immune activation and inflammatory fibrosis associated with SIV and HIV infection that is a significant factor in CD4^+^ T cell loss and incomplete immune reconstitution with ART. Serial access to these samples will also permit evaluation of early virological and immunological events during the eclipse phase of infection in primary draining LN and changes in immune responses including viral kinetics in response to infection, vaccination or therapies and evaluation of a critical viral reservoir site that harbors latent virus that has previously been possible only via major surgery or necropsy. Taken together, this procedure could lead to new insights into the effects of vaccines, therapeutics and latency reversal agents on mucosal immune responses and the viral reservoir. These results have significant implications for improving the study of HIV pathogenesis, vaccination and cure.
