# Correction: Laparoscopic Technique for Serial Collection of Para-Colonic, Left Colic, and Inferior Mesenteric Lymph Nodes in Macaques

**DOI:** 10.1371/journal.pone.0190764

**Published:** 2018-01-02

**Authors:** Jeremy Smedley, Rhonda Macalister, Solomon Wangari, Mercy Gathuka, Joel Ahrens, Naoto Iwayama, Drew May, Debbie Bratt, Megan O’Connor, Paul Munson, Michael Koday, Lifson, Deborah Heydenburg Fuller

The following information is missing from the Funding section: This project has been funded in part with Federal funds from the National Institute of Allergy and Infectious Diseases, National Institutes of Health, Department of Health and Human Services, under CEIRS Contract No. HHSN272201400005C.
